# Nutrition and Food Insecurity Screening in a Prescription Produce Program

**DOI:** 10.1093/geroni/igaf122.2385

**Published:** 2025-12-31

**Authors:** Katherine Falls, Ana Diallo, Kimberly Battle, Rachel Regal, Natalie Mansion, Genevieve Beaird, Johnathan Williams, Leland Waters

**Affiliations:** Virginia Commonwealth University, Richmond, Virginia, United States; Virginia Commonwealth University School of Nursing, Richmond, Virginia, United States; Virginia Commonwealth University, Richmond, Virginia, United States; Virginia Commonwealth University, Richmond, Virginia, United States; Virginia Commonwealth University, Richmond, Virginia, United States; Virginia Commonwealth University, Richmond, Virginia, United States; Virginia Commonwealth University Center for Translational Research, Richmond, Virginia, United States; Virginia Commonwealth University, Richmond, Virginia, United States

## Abstract

Understanding the complex social drivers that impact access to nutritious foods and dietary behaviors is essential to inform interventions that ultimately improve access to healthier options for older adults experiencing food and nutrition insecurity. The Prescription Produce Plan (PPP) is a Food is Medicine intervention embedded in the Mobile Health and Wellness Program, a wellness program serving low-income older adults. The PPP offers 6 goal-setting sessions and bags of fresh fruits and vegetables weekly or bi-weekly. The goal-setting sessions focus on behavior change to support chronic disease self-management. We conducted a pre-post analysis to measure the effects of the PPP on nutrition security and baseline descriptives of food security and access. Eighty-one participants enrolled, and 71 (88%) completed the program. On average, participants were 67 years old and reported five chronic diseases, with the most prevalent being hypertension (75%), type 2 diabetes (38%), and hypercholesterolemia (36%). At baseline, 43 participants (53%) screened positive for food insecurity, while 51% reported nutrition insecurity and 55% reported using food pantries to access groceries. The program was significantly associated with an 18% decrease in nutrition insecurity at the post-test (Z= -2.4, p = 0.02). These findings suggest that the PPP effectively addresses nutrition insecurity among older adults, emphasizing the importance of community-based interventions in tackling food access challenges. Additionally, using nutrition and food insecurity screening tools provides a deeper understanding of the complex nature of food access for older adults with significant health inequities.

